# GSDMD promotes neutrophil extracellular traps via mtDNA-cGAS-STING pathway during lung ischemia/reperfusion

**DOI:** 10.1038/s41420-023-01663-z

**Published:** 2023-10-04

**Authors:** Chen Zhao, Fangte Liang, Mengling Ye, Siyi Wu, Yi Qin, Lu Zhao, Lu Zhang, Jing He, Liming Cen, Fei Lin

**Affiliations:** 1 Department of Anesthesiology, Guangxi Medical University Cancer Hospital, Nanning, China; 2 Guangxi Clinical Research Center for Anesthesiology, Nanning, China; 3 Guangxi Engineering Research Center for Tissue & Organ Injury and Repair Medicine, Nanning, China; 4 Guangxi Health Commission Key Laboratory of Basic Science and Prevention of Perioperative Organ Disfunction, Nanning, China; 5 Department of Experimental Research, Guangxi Medical University Cancer Hospital, Nanning, China

**Keywords:** Acute inflammation, Cell death

## Abstract

Lung ischemia/reperfusion injury (LIRI) is a complex pathophysiological process, with the histopathological hallmark of neutrophils migrating into the lungs. Neutrophil extracellular traps (NETs) have been suggested to exert a critical role in the pathogenesis of inflammation and infection in humans and animals, while the exact functions and underlying mechanisms of NETs in LIRI remain insufficiently elucidated. In this study, we investigated the role of pore-forming protein gasdermin D (GSDMD) on NETs release in LIRI induced by lung ischemia/reperfusion (I/R). We found that disulfiram, a GSDMD inhibitor, dramatically reduced NETs release and pathological injury in lung I/R in vivo and in vitro. Additionally, GSDMD caused mitochondrial DNA (mtDNA) leaking into the neutrophil cytosol, and then the cytoplasmic mtDNA activated the cGAS-STING signaling pathway and stimulated NETs formation in lung I/R. Furthermore, inhibition of cGAS/STING pathway could inhibit cytosol mtDNA mediated NETs formation.

## Introduction

Lung ischemia/reperfusion injury (LIRI) is a significant complication during surgical procedures when oxygen supply to the lung has been decreased followed by a period of reperfusion. Primary graft dysfunction is the most severe outcome of lung ischemia/reperfusion (I/R) injury, exhibiting a robust correlation with elevated mortality [1]. Lung I/R can cause inflammation, noncardiogenic lung edema, and severe hypoxemia, which are all indicative of a disrupted function of the alveolar-capillary barrier [2, 3]. Nevertheless, the underlying mechanisms responsible for LIRI have not been comprehensively elucidated.

Neutrophil Extracellular Traps (NETs) are generated and secreted by activated neutrophils, and consist of web-like structures composed of modified decondensed chromatin and granule proteins. These granule proteins, such as myeloperoxidase (MPO) and neutrophil elastase (NE), have been identified as components of the neutrophil’s antimicrobial mechanism [4]. The strong inflammatory reactions are linked with neutrophil recruitment and infiltration in the lung, followed by NETs release [5, 6]. NETs were also implicated in a variety of noninfectious conditions in peripheral tissue injury [7]. In I/R-induced liver and intestinal injury, NETs inhibition would be the effective way in preventing and diminishing clinical symptoms [8, 9]. However, the regulation mechanisms of NETs in LIRI are poorly defined.

GSDMD, a member of the Gasdermin family, is cleaved by inflammasomes, generating a GSDMD-N fragment to be involved in the pore-forming and the release of IL-1β in pyroptosis [10]. Accumulating evidence has underscored particular functions of GSDMD in neutrophils, such as involving in canonical autophagy to secret IL-1β [11] and mediating NETs in transfusion-related acute lung injury and SARS-CoV-2 infection [12]. While others have reported that GSDMD-deficient neutrophils increase the formation of NETs in response to classical or non-classical inflammatory stimulation [13], whether GSDMD regulates the formation of NETs in LIRI is still unknown.

Cyclic GMP-AMP (cGAMP) synthase (cGAS) functions as a cytosolic DNA sensor, which can be stimulated through binding DNA, comprising mitochondrial DNA (mtDNA), nuclear DNA (nDNA), and DNA from bacteria [14]. cGAS catalyzes the biosynthesis of cGAMP, which acts as a second messenger to stimulate the adapter protein STING, leading to the induction of an inflammatory response [15]. Studies have verified the connections between GSDMD and the cGAS-STING pathway [16, 17]. Others reported NETs were highly enriched in mtDNA in adult-onset Still’s disease [18] and lupus-like disease [19]. However, there remains a lack of studies to reveal whether the cGAS-STING pathway is involved in lung I/R.

In this study, we aimed to explore whether GSDMD was engaged in NETs formation, and disulfiram (DSF), an inhibitor of GSDMD, was used to reveal whether GSDMD regulated NETs formation via the mtDNA/cGAS/STING pathway during LIRI.

## Results

### NETs were detected in lung I/R and neutrophil hypoxia/reoxygenation (H/R)

**Fig. 1:**
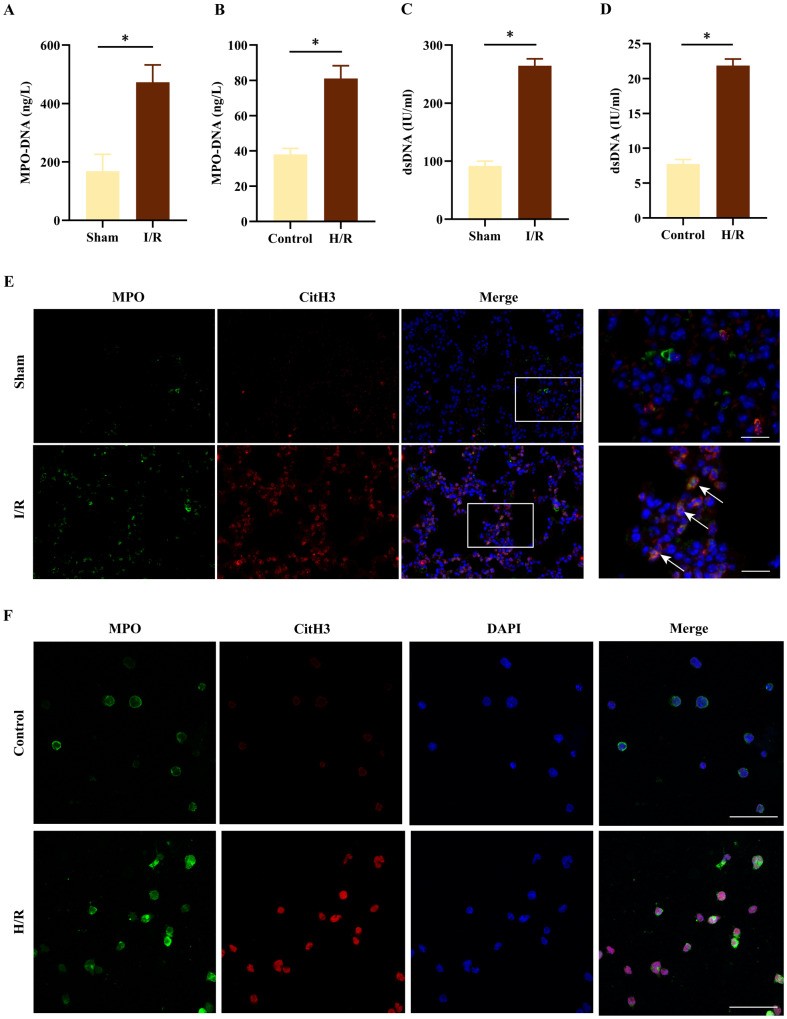
Lung I/R and neutrophil H/R induce NETs formation in vivo and in vitro. **A**, **B** MPO-DNA complex levels in BALF and neutrophil culture supernatants (*n* = 6). **C, D** Levels of dsDNA in BALF and neutrophil culture supernatants (*n* = 6). **E** Representative Immunofluorescence images of NETs stained for myeloperoxidase (MPO, green), citrullinated histone H3 (citH3, red), and DNA (DAPI, blue) in lung tissues (*n* = 3). Scale bar: 20 μm. **F** Representative Immunofluorescence images of NETs stained for myeloperoxidase (MPO, green), citrullinated histone H3 (citH3, red), and DNA (DAPI, blue) in neutrophils (*n* = 3). Scale bar: 50 μm. The data were expressed as mean ± SEM, *P < 0.05.

To investigate the role of NETs in the pathogenesis of lung I/R injury, we established a lung I/R model in mice. Compared to the sham group, the I/R group had elevated levels of both MPO-DNA (Fig. 1A) and double-stranded DNA (dsDNA) (Fig. 1C) in BALF. Then, we isolated neutrophils from healthy donors and evaluated the purity of neutrophils through Giemsa staining and flow cytometry. Neutrophils were clearly visible under the microscope, with a distinctive nucleus containing 2–5 lobes (Supplementary Fig. S1A). Flow cytometry analysis revealed that the neutrophil purity was over 97% (Supplementary Fig. S1B). Furthermore, the levels of MPO-DNA complexes and dsDNA were found to be elevated in the neutrophils subjected to H/R treatment (Fig. 1B, D). To better visualize NETs formation, we performed immunofluorescence staining for citrullinated histone H3 (citH3) and MPO on both lung tissue sections and isolated neutrophils. The results showed a significant increase in the colocalization of citH3 and MPO following I/R or H/R stimulus (Fig. 1E, F). Taken together, our findings demonstrated that I/R and H/R induction led to the formation of NETs both in vivo and in vitro.

### Inhibition of GSDMD suppressed H/R-induced NETs and mitochondrial dysfunction

**Fig. 2:**
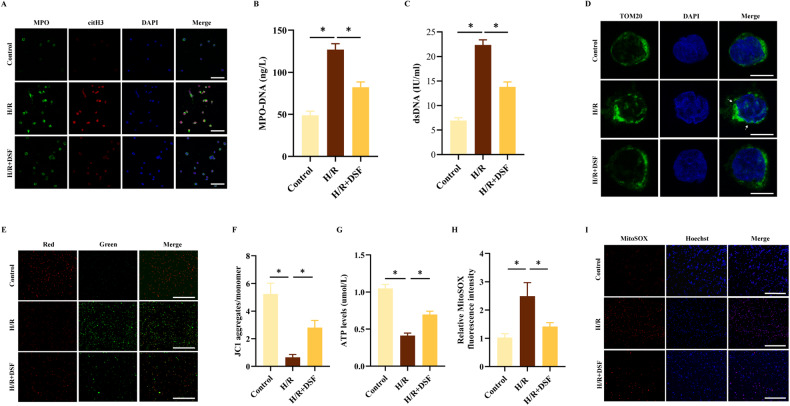
Inhibition of GSDMD suppresses NETs formation and mitochondrial dysfunction. Neutrophils were treated with DSF (30 μM) for 1 h before H/R. **A** Representative Immunofluorescence images of NETs stained for MPO (green), citH3 (red), and DNA (DAPI, blue) in neutrophils. Scale bar: 50 μm. **B**, **C** Levels of MPO-DNA and dsDNA in neutrophil culture supernatants. **D** Representative images of immunofluorescence co-staining of Mitochondria (anti-TOM20; green) and Nuclei (DAPI; blue) in neutrophils. Scale bar: 10 μm. **E**, **F** Representative Immunofluorescence and the ratio of JC-1 staining (red, J-aggregates; green, monomer) in neutrophils. Scale bar: 200 μm. **G** The amount of ATP. **H**, **I** MitoSOX fluorescence staining and its fluorescence intensity. Scale bar: 200 μm. The data were expressed as mean ± SEM (*n* = 3), *P < 0.05.

To investigate the role of GSDMD in NETs formation in vitro via the H/R model, we pre-treated neutrophils with DSF (30 μM) for 1 h before subjecting them to H/R stimulation. We found that the formation of NETs was markedly suppressed in the DSF-treated group compared to the H/R group (Fig. 2A–C). Additionally, the H/R group showed significantly increased fragmented mitochondria (Fig. 2D) and MitoSOX fluorescence intensity (Fig. 2H, I), reduced mitochondrial membrane potential (Fig. 2E, F) and ATP production (Fig. 2G) compared to the control group. Importantly, we observed that inhibition of GSDMD by DSF significantly decreased the count of fragmented mitochondria, mitochondrial membrane permeability, and the production of mitochondrial ROS and ATP, while also increasing the length of mitochondrial in H/R neutrophils (Fig. 2D–I). These findings suggested that treatment with DSF played a critical role in attenuating the progression of NETs, mitigating the disruption of mitochondrial homeostasis, and was associated with mitochondrial dysfunction induced by LIRI in vitro.

### GSDMD induced mtDNA leakage into the cytosol in H/R neutrophil

**Fig. 3:**
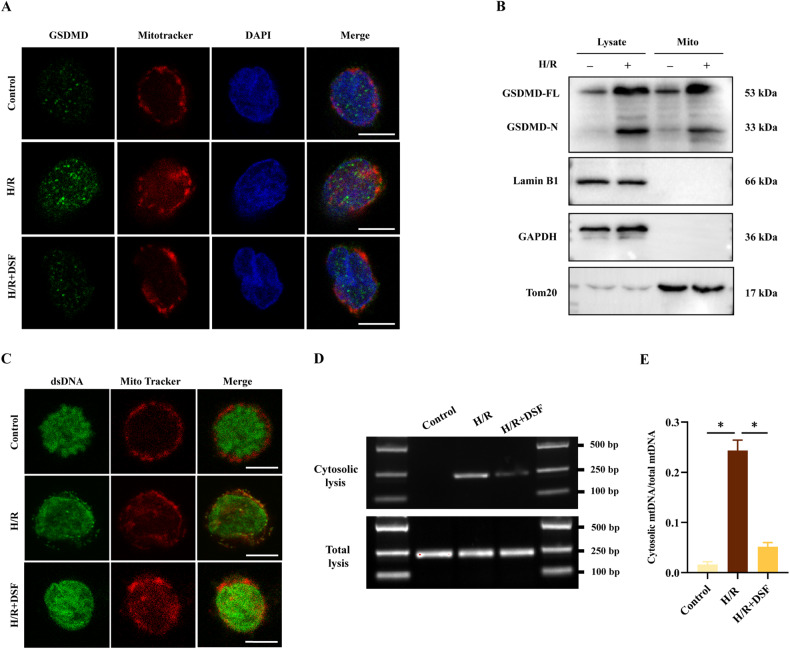
GSDMD induces mtDNA leakage into the cytosol in H/R neutrophils. **A** Representative Immunofluorescence images of GSDMD (green), mitochondria (red), and DAPI in the three groups. Scale bar: 10 μm. **B** Neutrophils were fractionated, whole-cell extracts and mitochondrion (Mito) were blotted using indicated antibodies. **C** Representative fluorescence images of dsDNA (green) and mitochondria (red) in neutrophils. Scale bar: 10 μm. **D** DNA agar gelatin electrophoresis images of mtDNAs in cytosolic lysis and total lysates of neutrophils. **E** RT-qPCR quantitative analysis of cytosolic mtDNAs normalized to mtDNAs of total lysates of neutrophils. The data were expressed as mean ± SEM (*n* = 3), *P < 0.05.

To reveal the mechanisms underlying the dysfunction of mitochondria in neutrophils, we examined the localization and activated form of GSDMD through immunofluorescence imaging and western blot analysis. Specifically, we utilized immunofluorescence microscopy to evaluate the colocalization of GSDMD (green, labeled by anti-GSDMD) and mitochondria (red, labeled by Mitotracker) following H/R stimulation. The results demonstrated a significant accumulation in the colocalization of GSDMD and mitochondria in the H/R group, which was markedly reduced after DSF treatment (Fig. 3A). Moreover, we found that the P31 GSDMD-N fragment localized in the mitochondria after H/R stimulation, but not in the control group (Fig. 3B). Next, we investigated whether the release of mtDNA was caused by GSDMD-mediated mitochondrial pore formation. The colocalization of dsDNA and mitochondria indicated that H/R-induced dsDNA outside of mitochondria was GSDMD-dependent, which was blocked by pre-treatment with DSF (Fig. 3C). To further validate the release of mtDNA was GSDMD-dependent, we extracted cytosolic DNA and analyzed it through agarose gel electrophoresis and real-time quantitative PCR (RT-qPCR) (Fig. 3D, E). The results showed that DSF treatment significantly decreased the levels of cytosolic mtDNA induced by H/R treatment, suggesting that leakage of mtDNA into the cytosol dependent on GSDMD. These findings suggested that GSDMD may be a critical factor in the regulation of mitochondrial homeostasis and mtDNA release following H/R-induced injury.

### Cytosolic mtDNA activated the production of NETs in H/R neutrophils

**Fig. 4:**
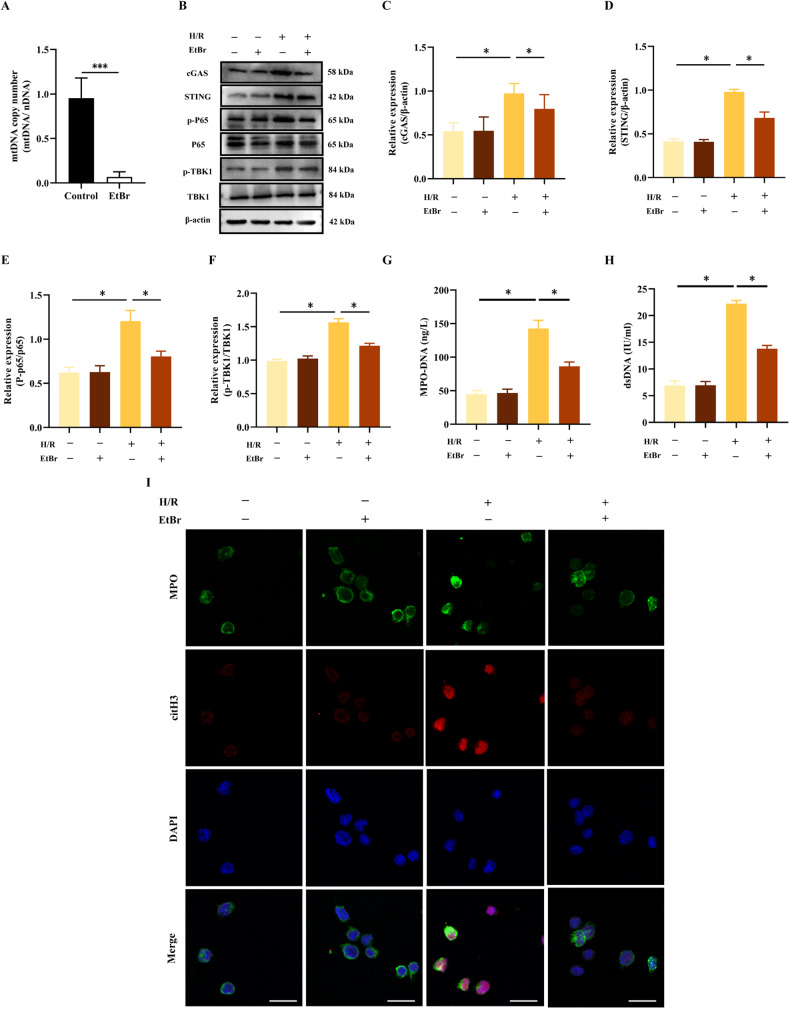
Cytosolic mtDNA activates the cGAS-STING signaling and the production of NETs in H/R neutrophils. **A** mtDNA copy number was assessed by quantitative PCR in neutrophils after treatment with the mtDNA inhibitor EtBr (1 μg/ml, 48 h). **B**–**F** Western blot analysis and quantification of the target protein normalized to total P65, total TBK1, and β-actin. **G**, **H** MPO-DNA and dsDNA levels in neutrophil culture supernatants. **I** Representative Immunofluorescence images of NETs stained for MPO (green), citH3 (red) and DNA (DAPI, blue) in neutrophils. Scale bar: 20 μm. The data were expressed as mean ± SEM (*n* = 3), *P < 0.05, ***P < 0.001.

To examine the effect of cytosolic mtDNA on NETs release, ethidium bromide (EtBr) was used to hinder the replication of mtDNA without affecting the replication of nDNA [20]. Following the administration of EtBr to neutrophils, mtDNA copy number was reduced by nearly 90% (Fig. 4A). We found that H/R-induced phosphorylation of NF-κB p65 and TBK1 was restricted in mtDNA-depleted neutrophils (Fig. 4B–F). Additionally, the depletion of mtDNA significantly decreased NETs formation induced by H/R treatment (Fig. 4G–I). These results implicated that cytosolic mtDNA could be deemed as a significant participant in NETs formation, which might be related with cGAS-STING pathways.

### The cGAS-STING pathway was necessary for the formation of mtDNA-induced NETs

**Fig. 5:**
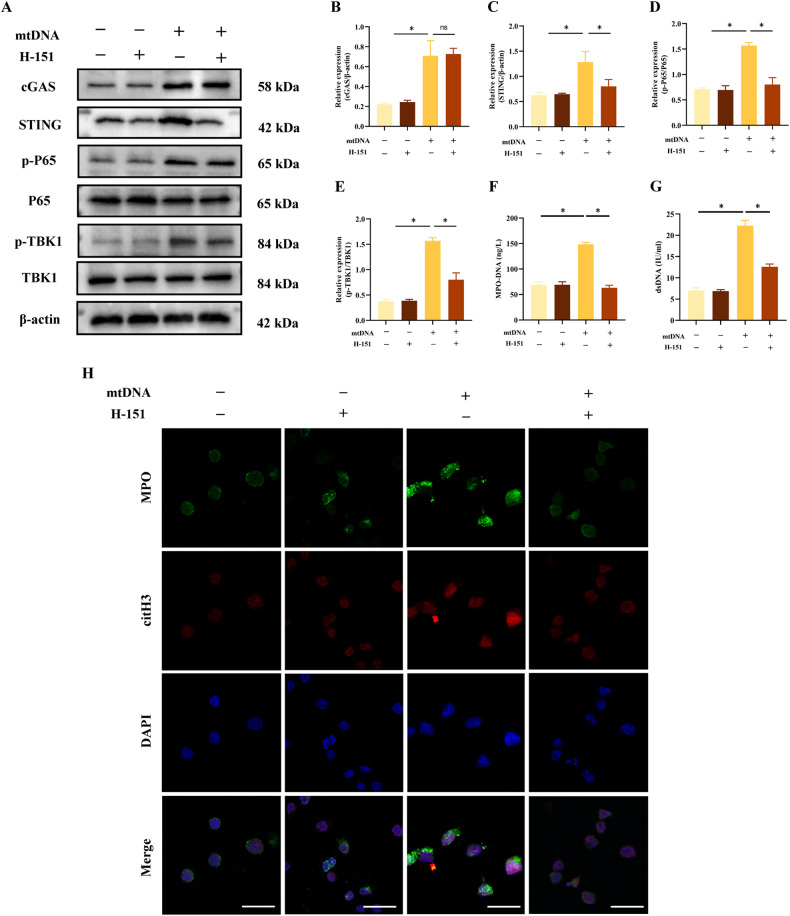
cGAS-STING pathway is necessary for the formation of mtDNA-induced NETs. Neutrophils were transfected with mtDNA (2 μg/ml,12 h) or treated with H-151 (5 Μm, 1 h). **A**–**E** Western blot analysis and quantification of the target protein normalized to total P65, total TBK1, and β-actin. **F**, **G** MPO-DNA and dsDNA levels in the supernatant of neutrophils. **H** Representative Immunofluorescence images of NETs stained for MPO (green), citH3 (red) and DNA (DAPI, blue) in neutrophils. Scale bar: 20 μm. The data were expressed as mean ± SEM (*n* = 3), ns not significant, *P < 0.05.

To verify whether cytosolic mtDNA triggers NETs formation via the cGAS-STING pathway, we isolated mtDNA and transfected it into neutrophils. H-151, a selective STING antagonist, was used to pharmacologically block the activity of STING. Western blot demonstrated that transfected cytosolic mtDNA induced cGAS-STING pathway activation, as evidenced by the phosphorylation of TBK-1 and NF-κB p65, which was abrogated by H-151 treatment (Fig. 5A–E). Similarly, neutrophils transfected with mtDNA produced more NETs than control neutrophils, which were inhibited by treating with H-151 (Fig. 5F–H). Overall, these findings suggested that cytosolic mtDNA promoted NETs formation via the cGAS-STING pathway in H/R neutrophils.

### Pharmacologic inhibition of GSDMD suppressed NETs release and lung inflammation in lung I/R

**Fig. 6:**
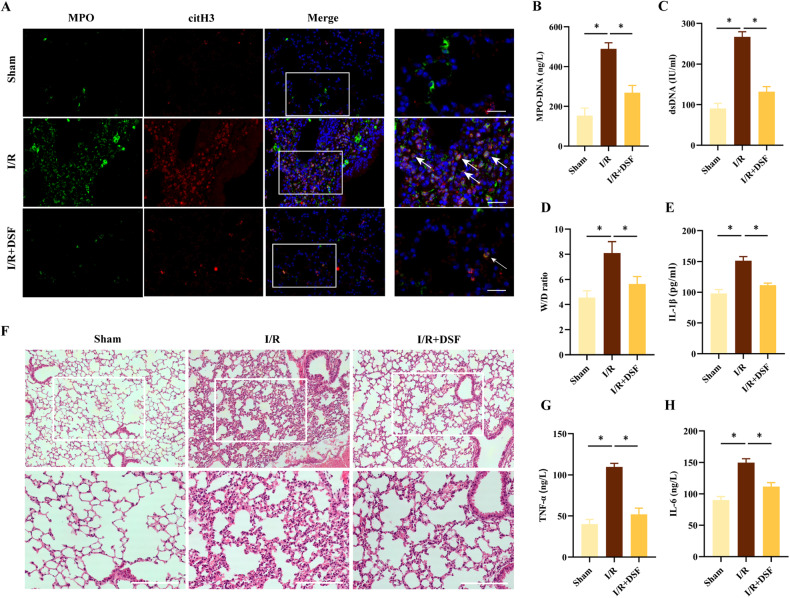
Inhibition of GSDMD inhibits the production of NETs and alleviates lung injury after lung I/R in vivo. Mice were treated with DSF (50 mg/kg, i.p.) 24 h and 4 h before surgery. **A** Representative Immunofluorescence images of NETs stained for MPO (green), citH3 (red), and DNA (DAPI, blue) in lung tissues (*n* = 3). Scale bar: 20 μm. Levels of MPO-DNA (**B**) and dsDNA (**C**) in BALF (*n* = 6). **D** W/D ratio of lung tissues (*n* = 6). Levels of IL-1β (**E**), TNF-α (**G**) and IL-6 (**H**) in BALF (*n* = 6). **F** H&E-stained lungs from 3 groups (*n* = 3). Scale bar:100 μm. The data were expressed as mean ± SEM, *P < 0.05.

To study the influence of DSF on lung I/R in vivo, C57BL/6 mice were given intraperitoneal injections of DSF (50 mg/kg) at 24 h and 3 h before the surgery. Similar to the in vitro findings, the in vivo results also demonstrated that pre-surgical DSF treatment decreased NETs formation induced by I/R (Fig. 6A–C). The results also showed DSF mitigated lung injury, which was characterized by reductions in wet/dry ratios (Fig. 6D), inflammatory cytokines (Fig. 6E, G, H) and lung tissue damage (Fig. 6F). Collectively, these results suggested that inhibition of GSDMD with DSF could protect mice from lung I/R and attenuate NETs release in pulmonary inflammation.

## Discussion

**Fig. 7:**
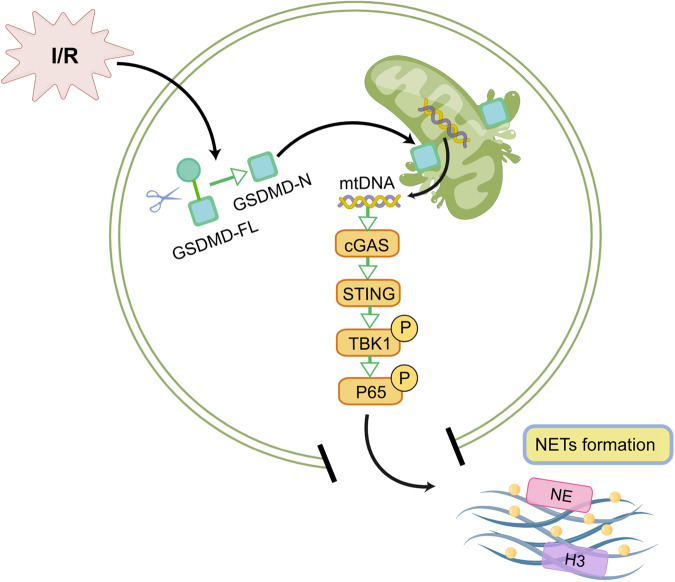
Proposed mechanism of GSDMD-induced NETs formation mediated by mtDNA-cGAS-STING pathway activation in LIRI (by Figdraw). Lung I/R promotes the cleavage of GSDMD into the active form GSDMD-N, resulting in mitochondrial dysfunction and mtDNA release into the cytosol of neutrophils. The cytosol mtDNA activates the cGAS-STING pathway, leading to producing NETs and lung injury.

This study aimed to investigate the involvement of the pore-forming protein GSDMD in the regulation of NETs release and the subsequent development of inflammation and injury during LIRI. By utilizing both in vivo and in vitro LIRI models, we demonstrated that pharmacologic inhibition of GSDMD with disulfiram prevented the release of NETs and reduced lung inflammation in LIRI mice. Importantly, our findings revealed a novel mechanism by which GSDMD mediate the release of mtDNA into the cytosol of H/R neutrophils, subsequently activating the cGAS-STING pathway and promoting NETs formation (Fig. 7).

NETs formation is triggered by the activation of protein-arginine deiminase 4, accompanied by the activation of MPO and NE, leading to chromatin decondensation [21, 22]. The nuclear envelope disintegrates to allow the release of chromatin into the cytoplasm, where it mingles with cytosolic proteins [23]. Finally, NETs are released outside the cell through the plasma membrane pores. NETs have been recognized as a double-edged sword in the innate immune response. On one hand, NETs play a crucial role in capturing and eliminating pathogens to protect against infection [24]. On the other hand, NETs have been demonstrated to induce pathology, such as impeding wound healing in diabetic patients [25] and exacerbating inflammation through interaction with platelets in sepsis [26]. Additionally, NETs contribute to renal dysfunction in I/R-induced acute kidney injury [27]. In this study, by measuring MPO-DNA complex and dsDNA levels, as well as the colocalization of citH3 and MPO both in vivo and in vitro, we observed a significant upregulation of NETs-bound components in the lungs and isolated neutrophils following lung I/R and neutrophil H/R. These results suggested that NETs participated in the process of LIRI.

Most studies have emphasized the role of cleaved GSDMD-N in forming plasma membrane pores in macrophages [20] and endothelial cells [28]. However, Mausita and colleagues reported that cleaved GSDMD-N could also produce pores in neutrophils, affecting intracellular organelles rather than the plasma membrane [11]. Another study showed that GSDMD could form pores in mitochondria, leading to the release of mitochondrial ROS and promoting a switch toward necroptotic cell death [29]. These findings suggested that GSDMD might play diverse roles in cell death and immune responses beyond its function in pyroptosis. Our study revealed that LIRI promoted the activation of neutrophil-GSDMD and provided evidence that NETs generation was a GSDMD-dependent process both in vivo and in vitro. Our findings supported the recruitment of GSDMD to mitochondria, where it formed pores via GSDMD-N. This process led to the release of mtDNA and a decrease in mitochondrial membrane potential, ultimately promoting mitochondrial injury. These results were consistent with a previous report demonstrating GSDMD ‘s ability to create mitochondrial pores and induce mtDNA release into the endothelial cell cytosol [30].

While NETs are mainly composed of chromosomal DNA, successive work have indicated that mtDNA can also be released with NETs in lupus-like disease [19, 31], suggesting a direct involvement of mtDNA in NETs formation. A Previous study has confirmed that cell-free mtDNA triggers NETs formation by the TLR9 pathway during lung transplantation [7] and by the cGAS-STING pathway in sickle cell disease [32]. However, these studies do not explore or elucidate an elective effect of cytosolic mtDNA in neutrophils on NETs formation. Here we discovered that the depletion of mtDNA decreased NETs formation and increased cytosolic mtDNA could stimulate NETs release in H/R neutrophils. These data indicated that cytosolic mtDNA had a pivotal role in NETs formation.

The release of mtDNA from necrotic cells can activate the cGAS-STING pathway [30], a well-known inflammatory mediator triggered by mtDNA in response to infection, cellular stress, and tissue damage, leading to the induction of NETs formation [33]. However, it remains unclear whether cytosolic mtDNA from active neutrophils can function as an intrinsic signal for activating the cGAS-STING pathway in LIRI. Our results demonstrated that H/R stimulation induced mtDNA release, followed by the activation of the cGAS-STING pathway. After the depletion of mtDNA, the phosphorylation of downstream signaling steps in the cGAS-STING pathway was alleviated. Notably, the transfected cytosolic mtDNA could activate the cGAS-STING pathway and subsequent NETs production, which were effectively restricted by the STING inhibitor H-151. These results strongly suggested that cytosolic mtDNA-dependent NETs formation was primarily mediated by its ability to activate cGAS-STING pathway.

DSF is originally used clinically as a drug to treat alcoholism, has been used for beyond 60 years and has not severe side effects. The last number of years has seen an explosion of interest in how DSF could inhibit pyroptosis by covalently modifying GSDMD [34]. In a recent study reported by Silva, they showed that reduced NETs production and alleviated inflammation via using disulfiram in septic mice [35]. Similarly, our results demonstrated that DSF inhibited NETs production, alleviated lung injury, and suppressed the inflammatory response both in vivo and in vitro.

In summary, we demonstrate that GSDMD plays a critical role in NETs release and lung injury via stimulating the release of mtDNA into the cytosol, followed by the activation of the cGAS-STING pathway during LIRI. Importantly, we prove that the GSDMD inhibitor DSF has pharmacological effects to improve LIRI outcomes.

## Materials and methods

### Animals

Male C57BL/6J mice, aged between 6 to 8 weeks, were purchased from the Animal Center of Guangxi Medical University (Nanning, China). These mice were provided with a pathogen-free environment and temperature-controlled room (24 °C) with access to standard food and water, within the animal facilities of Guangxi Medical University. Animals were fasted for 24 h before experiments and given free access to water. All procedures for mice were approved by the Guangxi Medical University Cancer Hospital Animal Research Committee.

### Murine LIRI model and experimental design

All mice were randomly allocated to three groups: the sham group (*n* = 12), the I/R group (*n* = 12), and the DSF (disulfiram, a GSDMD inhibitor) + I/R group (*n* = 12). Mice in the DSF + I/R group were given intraperitoneal injections of disulfiram (50 mg/kg) (PHR1690, Sigma-Aldrich, St. Louis, USA) at 24 h and 4 h prior to the surgery. The LIRI model was established as previously described [36]. Mice were anesthetized with an intraperitoneal injection of pentobarbital (50 mg/kg). After orotracheal intubation, mice were ventilated using a rodent ventilator (TOPO, Kent Scientific, South Elgin, USA) throughout the procedure. Mice underwent a left thoracotomy, followed by the interruption of the left pulmonary hilum via a microvascular clamp. Subsequently, the clamp was removed following 60 min of left lung ischemia, initiating a 6-h period of lung reperfusion. At the end of the experiment, all mice were humanly euthanized via cervical dislocation. After that, bronchoalveolar lavage fluid (BALF) and lung tissues were collected for subsequent experiments.

### Cytokine measurements and histology

BALF derived from the left lung was gathered using 1 ml of Hank’s Balanced Salt Solution (HBSS), and then the tumor necrosis factor (TNF)-α, IL-1β, IL-6, NET-associated MPO-DNA complexes and dsDNA levels were detected by ELISA kits (Elabscience Biotechnology, Wuhan, China). The lungs were fixed in paraformaldehyde (PFA), then embedded in paraffin. Tissue blocks were sectioned into 5-μm-thick slices, followed by HE staining for histological examination via a light microscope (ZEISS Axioscope 5).

### Lung wet/dry ratio

Lung tissues were extracted and the blood on the surface were gently wiped off with gauze. The wet weight was then measured. Then, the lung tissues were incubated at 60 °C for 72 h and weighed again to determine the dry weight. The wet/dry ratio was calculated to quantify the severity of pulmonary edema.

### Immunofluorescence staining

The lung tissue sections were dewaxed and rehydrated, then permeabilized with 0.1% Triton X-100 and blocked with 3% bovine serum albumin for 1 h. The sections were incubated with anti-MPO (sc-52707, Santa Cruz, CA, USA) and anti-citH3 (ab5103, Abcam, Cambridge, UK) antibodies overnight at 4 °C, followed by incubation with the fluorescent secondary antibody for 1 h in the dark. Sections were stained with DAPI solution for 5 min. After being washed with PBS three times, the sections were visualized and recorded using a fluorescence microscope (Nikon digital sight DS-FI2, Japan).

Neutrophils derived from peripheral blood were grown on coverslips pre-coated with 0.1% Poly-L-Lysine for 4 h in the lower chamber, then were fixed with 4% PFA for 10 min, permeabilized with 0.2% Triton X-100 for 10 min. After that, they were incubated with primary antibodies: anti-MPO, anti-citH3, and anti-TOM20 (sc-17764, Santa Cruz, CA, USA) overnight at 4 °C, followed by incubation with the corresponding fluorescent secondary antibody (Donkey Anti-Rabbit IgG H&L (Alexa Fluor® 594) (ab150076, Abcam, Cambridge, UK) and Goat Anti-Mouse IgG H&L (Alexa Fluor® 488) (ab150113, Abcam, Cambridge, UK) in the dark. DAPI was used to stain nuclear. Images were captured utilizing a confocal microscope (Leica TCS SP8, Leica, Germany).

### Isolation and treatment of blood neutrophils

Neutrophils were isolated from peripheral blood of healthy donors. Briefly, blood was mixed with Neutrophil Isolation lysis buffer (LZ11131, Haoyang Biotechnology, Tianjin, China) and centrifuged at 600 g for 30 min at room temperature. After centrifugation, the cell layer from the lower annular milky white gradient was collected as the neutrophil fraction. The purity of neutrophils was evaluated using a combination of flow cytometry with CD16-positive staining and Wright-Giemsa staining.

The neutrophils were cultured in RPMI 1640 medium supplement with 10% FBS at 37 °C and 5% CO_2_. A H/R model in vitro was established using neutrophils as described previously [36]. Briefly, neutrophils were cultured in glucose- and serum-free medium and equilibrated with 1% O_2_, 94% N_2_, and 5% CO_2_ for 2 h. For reoxygenation, Hypoxia-treated cells were incubated with glucose-containing medium at 37 °C in a 5% CO_2_ atmospheric air for 6 h. For drug treatments, DSF (30 μM) or the STING inhibitor H-151(5 μM, HY-112693, MedChemExpress, NJ, USA) were administered to the neutrophils 1 hour prior to hypoxia exposure. To deplete mitochondrial DNA, ethidium bromide (EtBr; 1 μg/ml, E7637, Sigma Aldrich, MO, USA) was administered for 48 h (40 h prior to H/R and 8 h during H/R). All the experiments were performed according to the Declaration of Helsinki and the Institutional Review Board of Guangxi Medical University.

### Measurement of ATP content

To determine neutrophil ATP levels, a bioluminescence assay kit (S0026, Beyotime, Shanghai, China) was used according to the manufacturer’s protocol. Briefly, the neutrophils were collected and lysed in the lysis buffer, then centrifuged at 12,000 × *g* for 5 min at 4 °C to collect the supernatant. ATP concentration in the samples was determined by luciferase assay reagent and analyzed using a microporous plate photometer (BioTek Instruments Inc, USA).

### Mitochondrial measurements

mitoSOX^TM^ Red mitochondrial superoxide indicator (M36008, Thermo Fisher, CA, USA) was utilized to quantify the levels of mitochondrial superoxide in neutrophils. Neutrophils were incubated with 5 µM mitoSOX for 10 min at 37 °C in the dark, and then washed with HBSS. Hoechst 33342 (C0030, Solarbio, Beijing, China) was used to stain the nuclei, and the images were viewed under a fluorescence microscope (Olympus BX43, Olympus, Tokyo, Japan).

For mitochondrial staining, MitoTracker ® Red CMXRos(200 nM, M7512, Thermo Fisher, CA, USA) was used following the manufacturer’s instructions. Cells were fixed and permeabilized for subsequent immunofluorescence staining.

### JC-1

JC-1 staining (C2006, Beyotime, Shanghai, China) was used to assess mitochondrial membrane potential. Cells were stained for 20 min at 37 °C and then washed in dye buffer. Subsequently, aggregates and monomers were measured at 490 nm/530 (ex/em) and 525 nm/590 nm (ex/em), respectively by fluorescence microscopy (Olympus BX43, Olympus, Tokyo, Japan).

### Mitochondria isolation

Mitochondria were isolated by utilizing the Cell Mitochondria Isolation Kit (C3601, Beyotime, Shanghai, China) per the manufacturer’s instructions. Neutrophils were solubilized in mitochondria isolation reagent with protease inhibitor, then incubated at 4 °C for 15 min, and the solution was centrifuged at 1000 × *g* for 10 min at 4 °C. The supernatant was then centrifuged at 12,000 × *g* for 10 min at 4 °C. The mitochondria were collected in the deposit and the supernatant containing mitochondria-free cytosolic fraction was carefully removed, followed by western blotting and RT-qPCR.

### Western Blot

Total protein extraction and quantification were performed on cell lysates maintained on ice. Protein lysates were separated by suitable SDS-PAGE gels and transferred to polyvinylidene difluoride membranes. The membranes were blocked with 5% skim milk in TBS-Tween, followed by incubating with the primary antibodies including GSDMD, Lamin B1, GAPDH, cGAS, STING, P65, β-actin (Proteintech, Wuhan, China), p-P65, TBK1, p-TBK1(Cell Signaling Technology, MA, USA) and TOM20 overnight at 4 °C. The PVDF membranes were washed three times with TBST and then incubated with the secondary antibody for 1 h at room temperature. Finally, the results were visualized via the Alpha Innotech System (BioRad) and analyzed by densitometry with Image Lab software.

### Measurement of mtDNA copy number and cytosolic mtDNA

Neutrophils with or without 1.0 μg/ml EtBr treatment and their total DNA were extracted by QIAamp DNA Micro Kit (56304, Qiagen, Hilden, Germany). 10 ng DNA was subjected to RT-qPCR analysis by SYBR Green Master Mixture (Vazyme, Nanjing, China). Relative quantification of mtDNA and nDNA was analyzed, with RNA-LeuUUR and β2-microglobulin serving as appropriate reference genes [37]. The mtDNA copy number was calculated depending on the mtDNA/nDNA ratio. The mtDNA primers were synthesized by Sangon Biotech (Shanghai, China) and the sequences were as follows: forward: 5′- CACCCAAGAACAGGGTTTGT-3′, reverse: 5′- TGGCCATGGGTATGTTGTTA-3′. The sequences of nDNA primers were as follows: forward: 5′- TGCTGTCTCCATGTTTGATGTATCT-3′, reverse: 5′- TCTCTGCTCCCCACCTCCAAGT-3′.

The cytosol of neutrophils was isolated by the Mitochondria Isolation Kit and the cytosolic fractions were collected without mitochondria. Approximately 50% of the neutrophils were subjected to cytosolic fractionation assay, while the remaining 50% were resuspended in whole-cell lysis buffer. MtDNA was purified from total cell lysis and cytosolic fractions, they were extracted using the QIAamp DNA Blood Mini Kit (51104, Qiagen, Hilden, Germany) following the manufacturer’s instructions. RT-qPCR was used to analyze the DNA, followed by agar gelatin electrophoresis to visualize the DNA abundance.

### mtDNA isolation and transfection

mtDNA was purified from neutrophils via the mitochondrial DNA isolation kit (ab65321, Abcam, Cambridge, UK) following the manufacturer’s instructions. Neutrophils were transfected with 2 μg/ml isolated mtDNA using the Lipofectamine 3000 (L3000150, Thermo Fisher Scientific, CA, USA) according to the manufacturer’s instructions.

### Statistical analysis

All results were shown as the mean ± SEM from independent experiments. Student’s *t* test (unpaired, two-tailed) was used to compare two groups, and one-way ANOVA was used to analyze the difference among multiple groups. *P* values < 0.05 was considered statistically significant.

### Supplementary information

Supplemental figure legend

supplemental figure

Original western blots

## Data Availability

The data used to support the findings of this study are available from the corresponding author upon reasonable request.
