# Supplementary material for: GSDMD promotes neutrophil extracellular traps via mtDNA-cGAS-STING pathway during lung ischemia/reperfusion
Source: Cell Death Discov. 2023 Oct 4;9:368. doi: 10.1038/s41420-023-01663-z (PMC10551007; doi:10.1038/s41420-023-01663-z)
Supplement: Supplementary file 1 — Supplemental figure legend [file 41420_2023_1663_MOESM1_ESM.docx]

**Figure Legend**

**Supplemental figure 1. Evaluation of the purity of isolated neutrophils from healthy donors.**

**A** Neutrophils were monitored by Wright-Giemsa staining and observed by microscopy. Scale bar:100 μm. **B** Flow-cytometric analysis of the expression of neutrophil marker CD16.
