# Supplementary figures and images for: GSDMD promotes neutrophil extracellular traps via mtDNA-cGAS-STING pathway during lung ischemia/reperfusion
Source: Cell Death Discov. 2023 Oct 4;9:368. doi: 10.1038/s41420-023-01663-z (PMC10551007; doi:10.1038/s41420-023-01663-z)

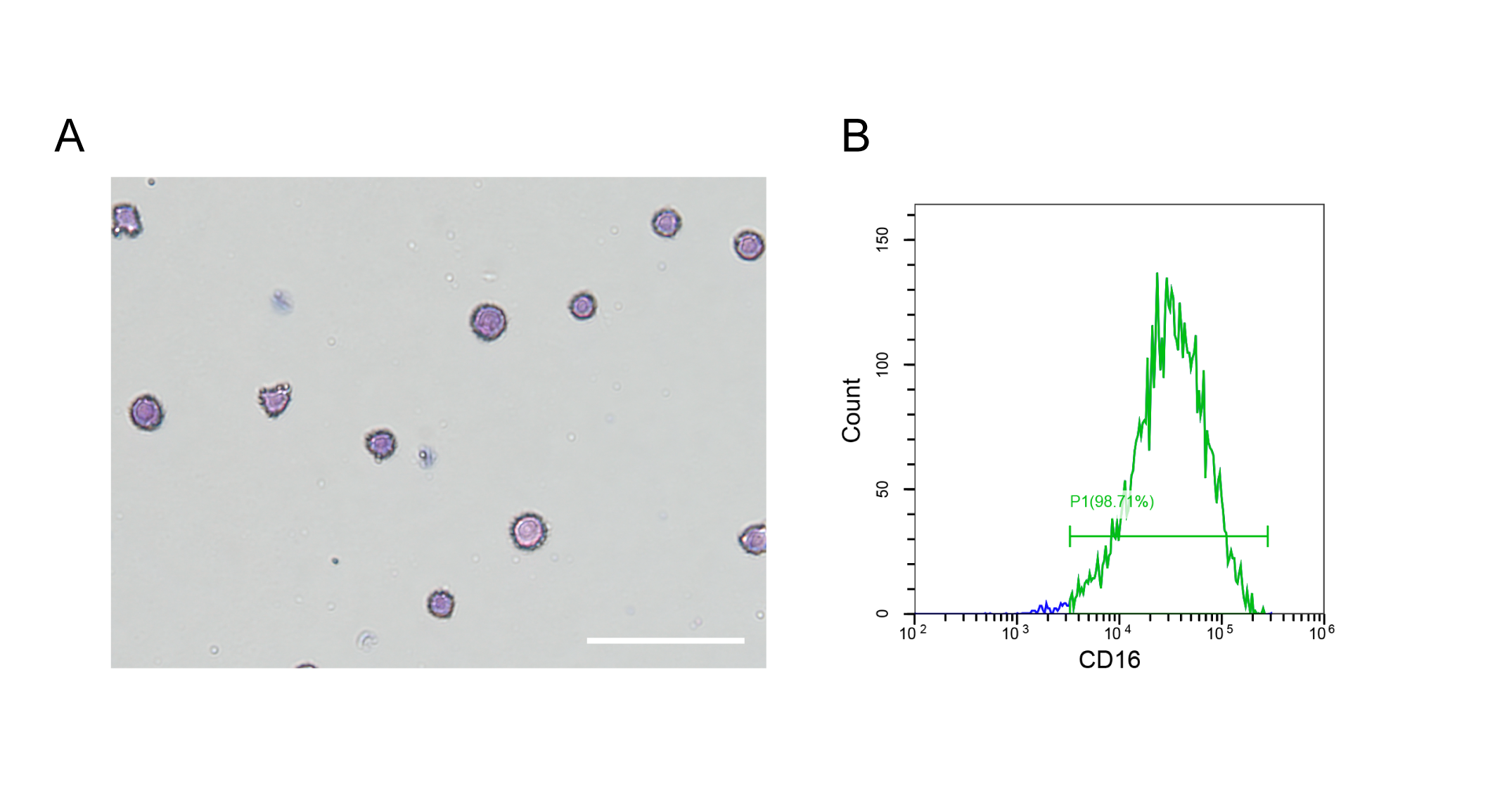

Supplement: Supplementary file 2 — supplemental figure [file 41420_2023_1663_MOESM2_ESM.tif]
