# Supplementary material for: GSDMD promotes neutrophil extracellular traps via mtDNA-cGAS-STING pathway during lung ischemia/reperfusion
Source: Cell Death Discov. 2023 Oct 4;9:368. doi: 10.1038/s41420-023-01663-z (PMC10551007; doi:10.1038/s41420-023-01663-z)
Supplement: Supplementary file 3 — Original western blots [file 41420_2023_1663_MOESM3_ESM.pptx]

## Slide 1
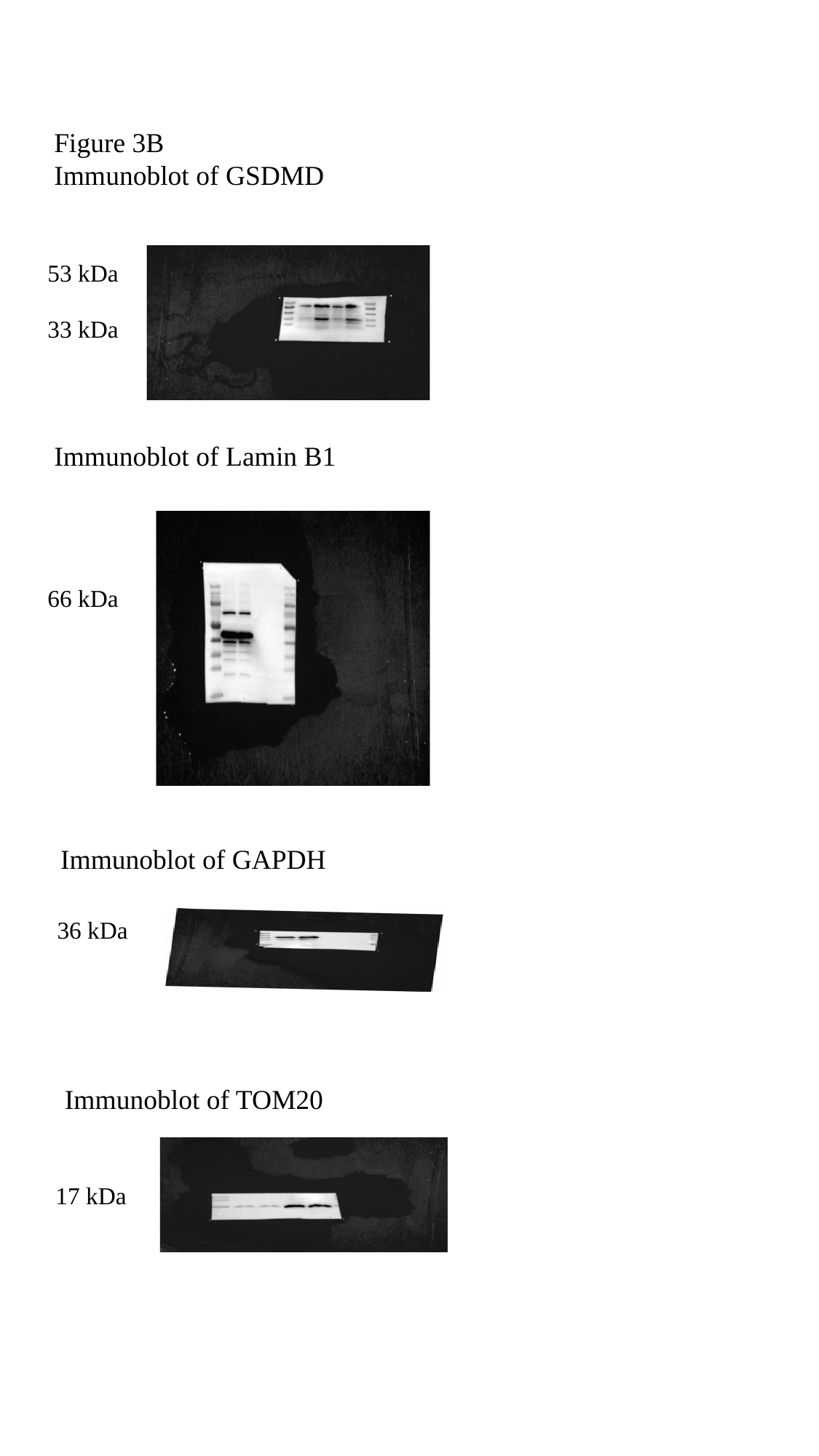

Figure 3B
Immunoblot of GSDMD
53 kDa
33 kDa
Immunoblot of Lamin B1
66 kDa
Immunoblot of GAPDH
36 kDa
Immunoblot of TOM20
17 kDa

## Slide 2
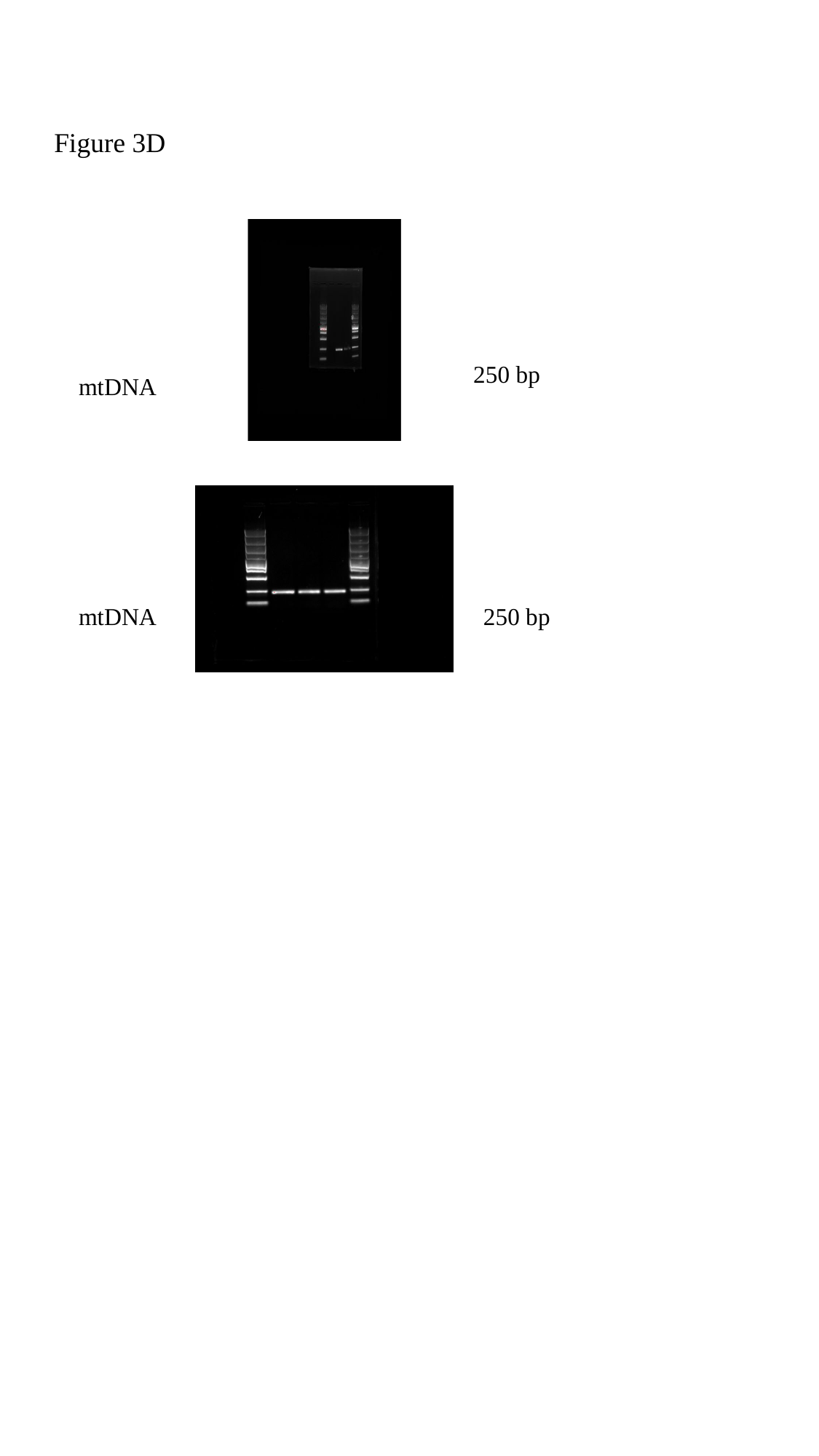

Figure 3D
250 bp
mtDNA
mtDNA
250 bp

## Slide 3
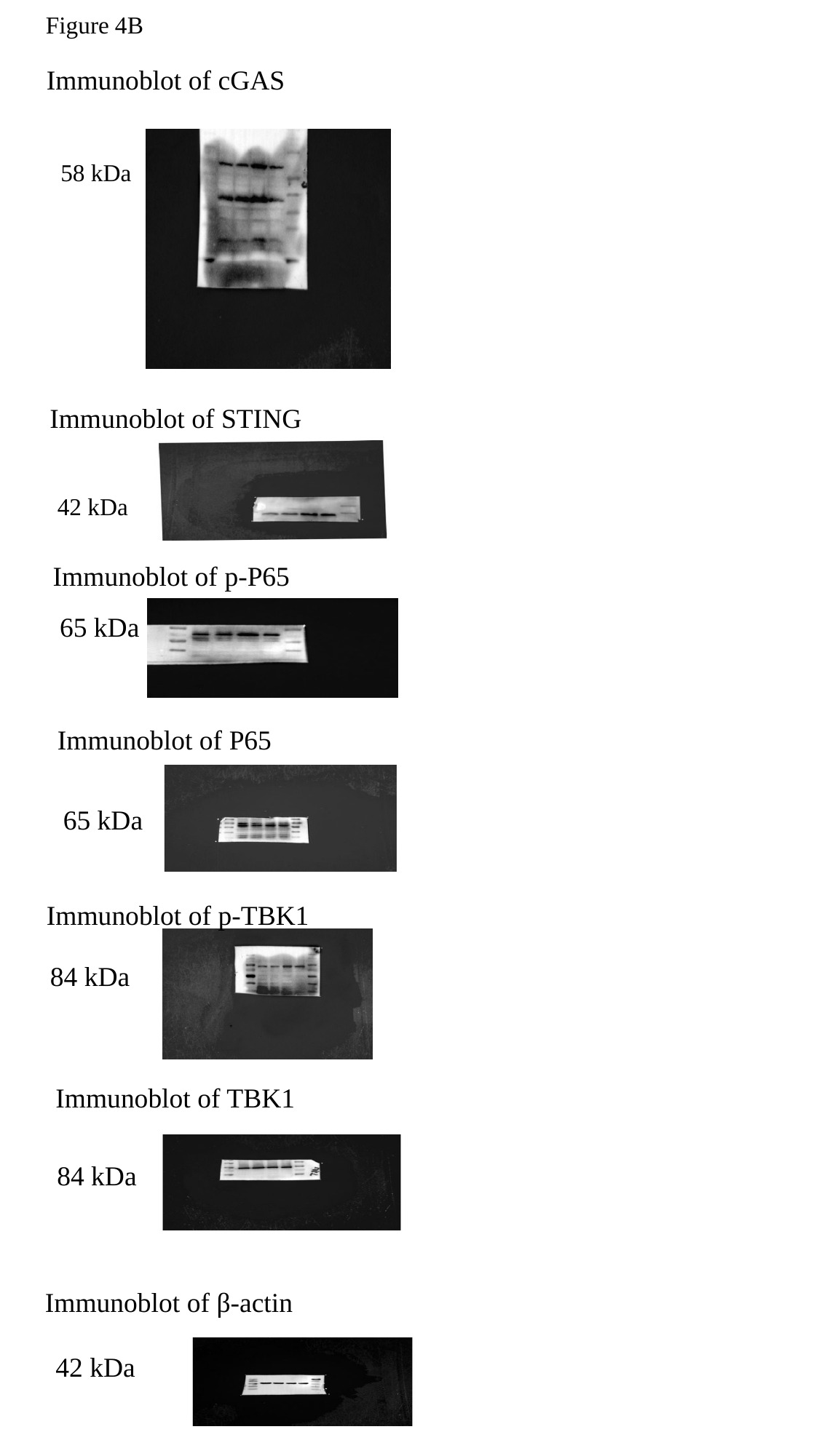

Figure 4B
Immunoblot of cGAS
58 kDa
Immunoblot of STING
42 kDa
Immunoblot of p-P65
65 kDa
Immunoblot of P65
65 kDa
Immunoblot of p-TBK1
84 kDa
Immunoblot of TBK1
84 kDa
Immunoblot of β-actin
42 kDa

## Slide 4
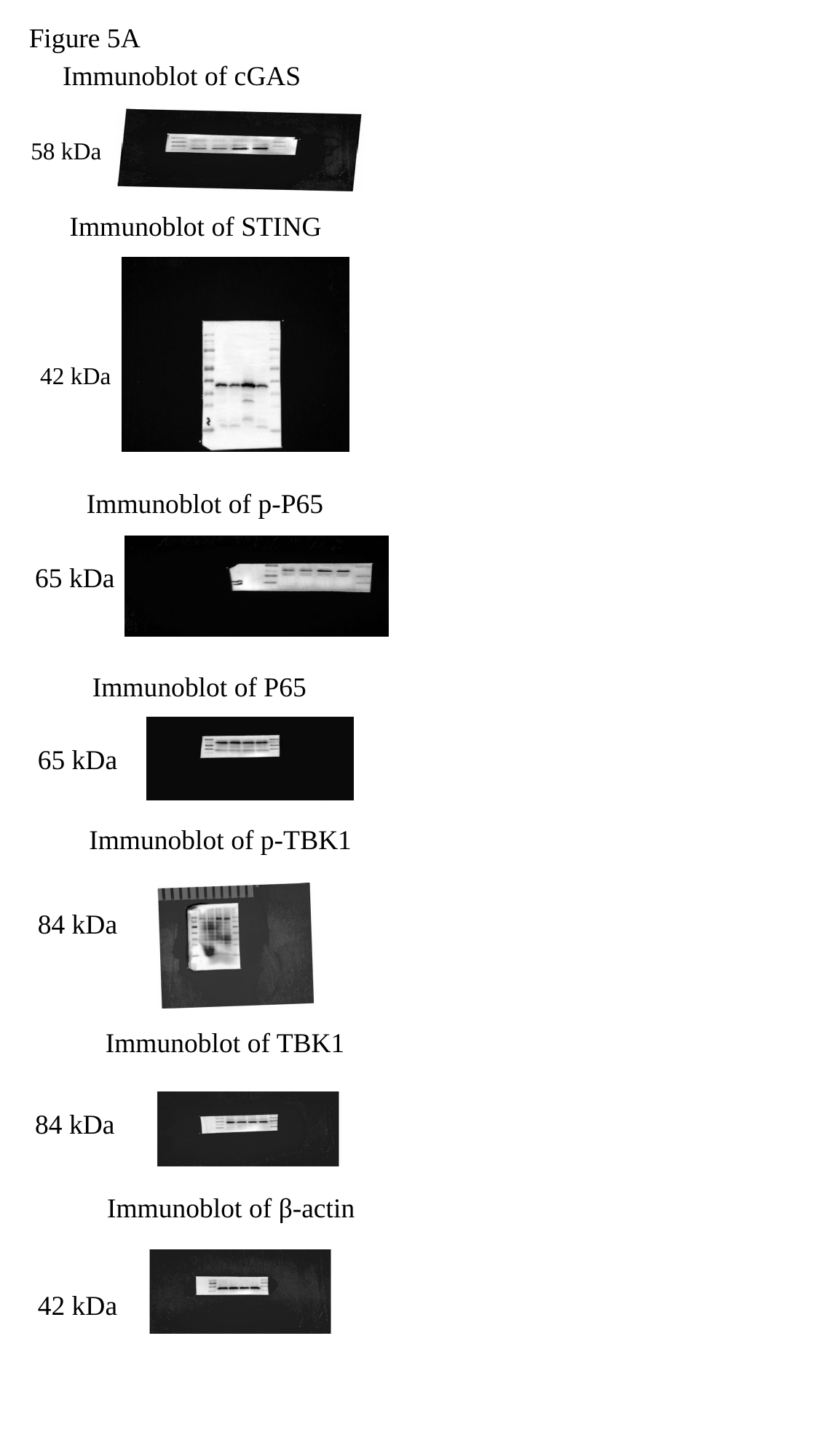

Figure 5A
Immunoblot of cGAS
58 kDa
Immunoblot of STING
42 kDa
Immunoblot of p-P65
65 kDa
Immunoblot of P65
65 kDa
Immunoblot of p-TBK1
84 kDa
Immunoblot of TBK1
84 kDa
Immunoblot of β-actin
42 kDa
